# Diabetes in pregnancy and offspring cardiac function: a systematic review and meta-analysis

**DOI:** 10.3389/fped.2024.1404625

**Published:** 2024-07-18

**Authors:** Camilla Bjørn Skovsgaard, Anna Møller, Jesper Vandborg Bjerre, Ulla Kampmann, Kasper Jacobsen Kyng

**Affiliations:** ^1^ Department of Paediatrics, Institute of Clinical Medicine, Aarhus University Hospital, Aarhus, Denmark; ^2^ Steno Diabetes Center Aarhus, Aarhus University Hospital, Aarhus, Denmark

**Keywords:** infants of mothers with diabetes, cardiac function, diabetes in pregnancy, echocardiography, systematic review

## Abstract

**Introduction:**

Diabetes in pregnancy is associated with impaired offspring cardiac function. The objective of this systematic review was to determine the effect of diabetes in pregnancy on cardiac function in the offspring measured by echocardiography.

**Methods:**

PubMed, Embase, Cochrane CENTRAL and Web of Science databases were searched from 1992 to June 27, 2023. Studies reporting offspring (age < 18 years) cardiac function by echocardiography compared between any type of diabetes in pregnancy and healthy control pregnancies were included. Study selection, quality assessment and risk of bias was independently performed by two reviewers. Meta-analyses was performed where possible.

**Results:**

Thirty-one observational studies were included 1,679 cases and 2,694 controls. In the first week of life (23 studies, *n* = 2,663), intraventricular septum diastolic diameter (hypertrophy) was increased, while myocardial performance index (global function) and LV E/A-ratio (diastolic function) were decreased. No difference was found for left ventricular ejection fraction (systolic function). At 1–6 months (4 studies, *n* = 454) studies found hypertrophy, and decreased global function, but no difference in systolic or diastolic function. At 1–8 years (7 studies, *n* = 1,609) no difference was found. The available data did not allow for sub-analysis based on the type of diabetes, treatment, or glycemic control.

**Conclusions:**

Diabetes in pregnancy is associated with cardiac hypertrophy and impaired global cardiac function in infants up to six months old. The few studies reporting on older children found no difference in the parameters investigated. Longitudinal studies employing more advanced echocardiographic measures or MRI are needed to evaluate consequences for long-term cardiac health.

**Systematic Review Registration:**

https://www.crd.york.ac.uk/, identifier (CRD42022312471).

## 1 Introduction

Maternal diabetes is associated with an increased risk of congenital cardiac malformations and myocardial hypertrophy but also functional myocardial changes with diastolic and systolic dysfunction (1, 2). The clinical presentation after birth ranges from asymptomatic to severely compromised with signs of congestive heart failure and respiratory distress (3). Symptomatic infants of mothers with diabetes are often screened with echocardiography, but changes in cardiac function may exist without immediate symptoms (4, 5). While the myocardial hypertrophy is usually transient with spontaneous resolution in the first months of life, it is not known to what degree functional cardiac changes persist. Children born to mothers with diabetes have increased rates of metabolic disease and early onset cardiovascular disease from childhood to early adulthood (6–9). The rate of cardiovascular complaints increases with the severity of maternal diabetic complications, e.g., poor glycaemic control, ketoacidosis, hyperlipidaemia, hypertension and/or maternal cardiovascular disease. This is increasingly important with the growing incidence of diabetes mellitus associated with the population aging, decreasing physical activity, urbanization, and an expanding obesity epidemic (10, 11). In Europe, pregestational diabetes (PDM) now affects about 1 percent of all pregnancies (12), and the prevalence of gestational diabetes (GDM) has increased from 5,4% in 2016 to 8,3% in 2021 (13, 14). More knowledge is needed to understand the consequences of altered cardiac function in the offspring, and to plan clinical follow-up for evaluation of the long-term effects of maternal diabetes and glycaemic control during pregnancy. Thus, the aim of this study was to systematically review current knowledge on the cardiac function assessed by echocardiography in infants and children of mothers with diabetes compared to healthy controls. Meta-analysis was performed where possible.

## 2 Methods

This review was made according to the Preferred Reporting Items for Systematic Review and Meta-Analysis (PRISMA) (15). The protocol was registered with PROSPERO International Prospective Register of Systematic Reviews (identifier CRD42022312471) (16).

### 2.1 Studies included and eligibility criteria

#### 2.1.1 Studies included

Peer reviewed studies in English, published from 1 January 1992 until 27 June 2023 were eligible for inclusion. Observational studies, except for case reports, reporting on one or more of the pre-specified outcomes were included.

#### 2.1.2 Population

Offspring of mothers with diabetes during pregnancy, either PDM or GDM, compared with healthy controls born to mothers without diabetes were eligible for inclusion. Exclusion criteria for both groups were infants with chromosomal abnormalities or congenital anomalies, any concomitant condition or disease in the offspring known to independently affect cardiac function, or age at outcome acquisition over 18 years.

#### 2.1.3 Exposure and comparators

Exposure was defined as any type of maternal diabetes (PDM including diabetes mellitus type 1 (T1DM) and type 2 (T2DM) or GDM), meeting common classification criteria, e.g., WHO guidelines (17), or American Diabetes Association (ADA) (18). Comparators were infants or children born to healthy women without diabetes.

#### 2.1.4 Outcome measures

The primary outcome was cardiac function assessed by echocardiography in six categories with associated example echocardiographic parameters (Table 1): (1) Left ventricular (LV) and right ventricular (RV) morphology, (2) Systolic function, (3) Diastolic function, (4) Global cardiac function, (5) Pulmonary hemodynamics, (6) Persistant ductus arteriosus (PDA).

**Table 1:** Cardiac function outcome measures.

Cardiac function category	Example echocardiographic parameters
1. Left ventricular (LV) and right ventricular (RV) morphology	Interventricular septal thickness diastole (IVSd), valve- and chamber dimensions
2. Systolic function	Left ventricular ejection fraction (LVEF), fractional shortening, strain and cardiac output
3. Overall cardiac function	Myocardial performance index (MPI) and cardiac torsion
4. Diastolic function	Left ventricular E/A-ratio (LV E/A), tissue velocities (E’, A’, S’) and strain
5. Pulmonary hemodynamics	Pulmonary artery pressure (PAP) and pulmonary artery acceleration time (PAAT)
6. Persistent ductus arteriosus (PDA)	PDA open or closed

The outcomes were listed in order of importance with respect to which are most likely to impact medical decision making and possibly future cardiac health. The primary outcome category of cardiac morphology including hypertrophy is not directly a measure of cardiac function, but is associated with and may be a driver of functional cardiac imparment in the context of diabetes in pregnancy. The rationale for the secondary categories was that some echocardiographic measures reflect mainly systolic or diastolic function, while others reflect global cardiac function. Pulmonary hemodynamics and PDA were included as maternal diabetes is associated with both an increased risk of persistant pulmonary hypertension of the newborn (PPNH), and of having a PDA.

#### 2.1.5 Subgroup analysis

Subgroup analysis was pre-planned based on (1) Type of diabetes, (2) Maternal glycaemic control (glycated haemoglobin, HbA1c levels), and (3) Offspring age at outcome acquisition.

### 2.2 Search strategy

The search strategy was developed with a medical librarian (19). We searched PubMed, Embase, Cochrane Database (CENTRAL) and Web of Science databases. For each database a specific search strategy was developed using subject headings and free text related to “cardiac function”, “infants”, “children”, and “diabetes”. The search was performed on June 27nd 2023. The complete search strategy is available as Supplementary Table S1.

### 2.3 Study selection and data extraction

Screening of titles and abstracts of the identified studies was done independently by two reviewers (CBS and AM). Disagreements were resolved by discussion, or if consensus was not reached, by consultation with a third reviewer (KJK).

Data from eligible studies was extracted using a modified data collection template based on the Cochrane Consumers and Communication Review Group Data Extraction Template (20). Two reviewers (CBS and AM) extracted data independently. Authors were not contacted for missing information.

The following data was extracted from included studies: title, first author, country, journal, publication year, conflicts of interest, study design, methodology, facility, study period, type of ultrasound equipment used, single or repeated outcome acquisition, recruitment, adjustment for confounders, number of cases and controls, in- and exclusion criteria, maternal characteristics (type and treatment of diabetes, HbA1c, body mass index, age at birth), and offspring characteristics (gestational age, birth weight, postnatal age at outcome acquisition, gender, co-morbidities).

### 2.4 Quality assessment

Quality assessment was performed by two reviewers (CBS and AM) independently using a modified version of the Newcastle-Ottawa Scale (NOS) specific for quality assessment of observational studies (20, 21). Disagreements were resolved by discussion. If no consensus was reached, a third reviewer (KJK) was consulted.

Based on their design, studies were divided into two groups (case-control, and cohort studies). Each study in the respective groups was awarded points in three categories for a maximum of nine points: (1) Selection (0–4 points) (2) Comparability (0–2 points) (3) Outcome/Exposure (0–3 points). Items in each category are listed in Table 2. Studies scoring 7–9 points were evaluated as high quality, 4–6 points as high risk of bias, and 0–3 points as very high risk of bias.

**Table 2:** Risk of bias using a modified Newcastle-Ottawa Scale (NOS) specific for quality assessment of observational studies.

Study	Selection	Comparability	Outcome/exposure	Overall score (0–9)
Case-control studies^a^				
Al-Biltagi et al. (2)	****	**	**	8
Arslan et al. (22)	***	*	**	6
Ghandi et al. (23)	****	**	**	8
Smith et al. (24)	****	**	**	8
Aguilera et al. (25)	***	**	***	8
Arslan et al. (26)	****	**	**	8
Çimen and Karaaslan (27)	****	*	**	7
Hăşmăşanu et al. (9)	****	*	**	7
Vela-Huerta et al. (28)	****	**	**	8
Bagheri et al. (29)	****	*	**	7
Blais et al. (6)	****	**	***	9
Falqui et al. (30)	***	**	**	7
Kozák-Bárány et al. (3)	****	**	**	8
Mehta et al. (31)	**	*	***	6
Schierz et al. (32)	****	**	**	8
Sobeih et al. (33)	****	**	**	8
Vela-Huerta et al. (34)	***	**	**	7
Vela-Huerta et al. (35)	****	*	**	7
Sonaglioni et al. (36)	****	**	**	8
Ergenc et al. (37)	****	**	**	8
Di Bernardo et al. (38)	****	**	**	8
Cohort studies^b^				
Iwashima et al. (1)	****	**	**	8
Do et al. (39)	****	*	**	7
Katheria and Leone (40)	****		**	6
Hoodbhoy et al. (41)	****	**	***	9
Lestari et al. (42)	****	*	***	8
Samanth et al. (5)	****	*	***	8
Zablah et al. (4)	****	**	**	8
Rijpert et al. (7)	***	**	**	7
Li et al. (43)	****	**	***	9
Jacquemyn et al. (44)	***	**	**	7

The symbols *, **, ***, and **** is a symbol of points given in the 3 categories: selection (0–4 points), comparability (0–2 points), and outcome/exposure (0–3 points). This is applicable in both group a (case/control studies) and group b (cohort studies).

^a^ NOS items case-control studies. Selection: case definition, case representativeness, selection of controls, definition of controls. Comparability: of cases and controls (0–2 points). Exposure: ascertainment of exposure, same method of ascertainment for cases and controls, non-responders.

^b^ NOS items cohort studies. Selection: representativeness of the exposed cohort, selection of the non exposed cohort, ascertainment of exposure, demonstration that outcome of interest was not present at start of study. Comparability: of cohorts (0–2 points). Outcome. Assessment of outcome, was follow up long enough for outcomes to occur, adequacy of follow up of cohorts.

### 2.5 Statistics and synthesis of results

Within each cardiac function category the most reported echocardiographic parameter was used for synthesis of results across studies. For the four most widely reported ultrasound parameters of cardiac function (IVSd, LVEF, MPI and LV E/A) in the first week of life outcomes were extracted as mean ± standard deviation (SD) for meta-analysis. Where no SD was reported, an average SD was imputed to enable inclusion in the meta-analysis. Where separate means and SDs were listed for subgroups of diabetes a combined data group was made for any type of diabetes by decomposing mean and SD (45, 46). Meta-analysis was performed in Review Manager 5.4.1 1 (The Cochrane Collaboration, 2020) using a random effect model and reported as mean differences between infants of mothers with any type of diabetes vs. infants of healthy control mothers. Between study heterogeneity was assessed using the I^2^ statistic (47). The heterogeneity of remaining results did not allow for meta-analysis. As no summary measure was applicable across all studies, we aggregated results for each cardiac function category. According to Popay et al (48), a tabulated and narrative summarisation of results was done, as in previous systematic reviews by our group and others (49, 50). Study results were summarized for each outcome grouped by age at outcome acquisition (first week of life, 1–6 months, or 2–8 years). Analysis was subgrouped by age group as the included outcome measures are known to change with age. Further, while myocardial hypertrophy is usually thought to be transient with spontaneous resolution in the first months of life, it is not known to what degree functional cardiac changes persist into later life and whether they influence the risk of early onset cardiovascular disease. Echocardiographic outcome assessments for the individual studies were categorized as decreased, increased or no difference. An overall conclusion was made for each outcome in each age group, based on the number of studies reporting an increase, decrease or no difference. If the number of studies was equally distributed among the categories, the highest number of cases determined the overall conclusion. Type of maternal diabetes was described. Treatment of diabetes and levels of HbA1c during pregnancy was summarized as average values and percentages. Publication bias was assessed graphically by funnel plots when 10 or more studies were eligible for meta-analysis (47).

### 2.6 Comparison to established normative values (Z-scores)

Comparing the absolute echocardiographic measurements (Supplementary Table S4) to established normative values (Z-scores) provides a context for interpreting the cardiac function of IDMs. For the purpose of generating example Z-scores we used, for the first week of life: 3.5 kg, 52 cm, BSA 0.23 m^2^; for 1–6 months of age: 6 kg, 60 cm, BSA 0.3 m^2^, for 1–8 years: 16 kg, 100 cm, BSA 0.66 m^2^. Based on these parameters example Z-score means (Z = 0) were retrieved from the “Cardio Z” App for IVSd (51) LV E/A (52) and MPI (53). First week of life: IVSd (3.7 mm), LV E/A (1.25), MPI (0.34); 1–6 months of age: IVSd (4.0 mm), LV E/A (1.24), MPI (0.34); 1–8 years: IVSd (5.1 mm), LV E/A (1,6), MPI (0.35). Normal pediatric LVEF is 56%–78%.

## 3 Results

### 3.1 Study characteristics

**Figure 1:**
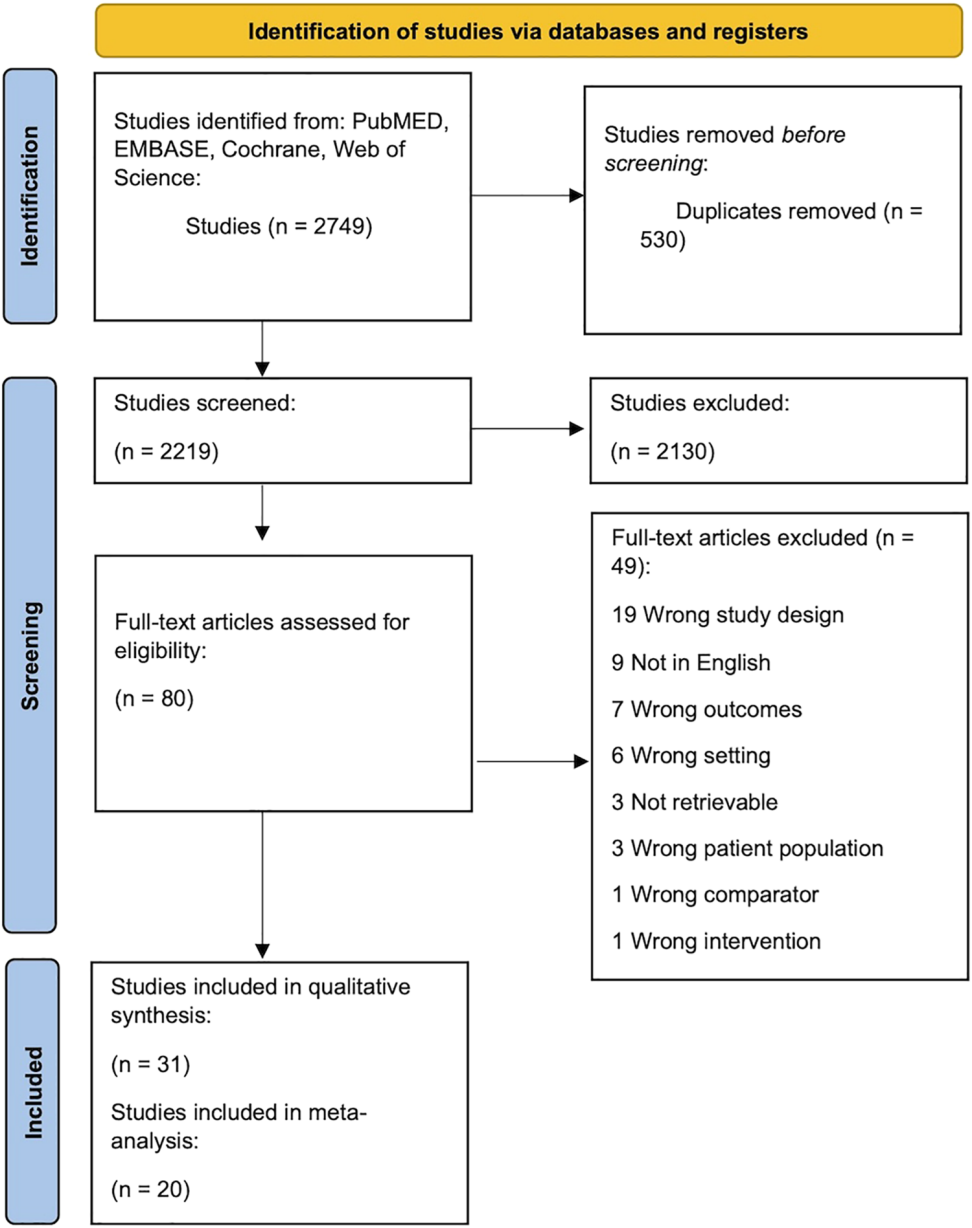
Flow diagram summarizing the screening and selection of studies for inclusion. After removal of duplicates, 1993 records were identified through database search. These were systematically screened leading to the inclusion of 31 publications with 20 studies eligible for meta-analysis (15).

We identified 31 studies which met the inclusion criteria and were eligible for inclusion in the review (Figure 1) (1–7, 9, 22–44). Ten studies were observational cohort studies with a control group, and 21 were case-control studies. In total, the studies reported on 1,679 diabetic pregnancies and 2,694 healthy control pregnancies. Six studies (5, 26, 27, 31, 36, 44) repeated measurements on the same cases and controls at different ages. Thus, the final dataset included 2023 echocardiographies from cases and 2,901 echocardiographies from healthy controls. The total number of cases and controls per study are presented in Supplementary Table S2.

Of all cases 100 women had T1DM, 28 had T2DM, 1,250 had GDM, and in 301 cases the type of diabetes was not described. Of the 1,679 diabetic mothers, 218 were treated with diet alone (12.5%), 66 were treated with oral anti-diabetic medication (3.8%), 227 were on insulin therapy (13.0%), and 16 received a combination of the three therapy methods (0.9%). For the remaining 1,152 women, diabetes treatment was not specified (69.7%). Two out of three studies reporting on oral anti-diabetic medicine reported the type of anti-diabetic medicine: metformin in 24 women (25), and glyburide in 17 women (4). Both groups of women treated with specific anti-diabetic medication had GDM. In 18 studies, glycaemic control was not reported as individual HbA1c levels, but defined as good control, poor control, or no control at all. One study reported HbA1c as a correlation with aortic stiffness (27). In the 12 remaining studies (3, 4, 7, 23, 25, 26, 28, 30, 32, 34, 36, 38) the median HbA1c in the diabetic women was 7.0% across studies. Data on diabetes treatment and levels of glycaemic control in the individual studies are presented in Supplementary Table S3.

### 3.2 Age at outcome assessment

Twenty-three studies performed echocardiographic measurements on infants in the first week of life (1–5, 9, 22–24, 26–38, 40). Four studies performed measurements in the first 1–6 months of life (5, 25, 26, 36). Seven studies performed measurements on children at the age of 2–8 years (6, 7, 39, 41–44). Three studies performed repeated measurements in the first week of life and at 1–6 months (5, 26, 36), and one study repeated measurements two times at 1–8 years (44). All extracted echocardiographic outcome measurements in each age group are presented in Supplementary Table S4.

### 3.3 Type of diabetes and diabetes classification criteria

ADA diabetes classification criteria were used in six studies for PDM and GDM (5, 28, 35, 37, 38, 43), NICE criteria were used in one study for GDM (25), and WHO criteria were used in three studies for GDM (2, 23, 42). Four studies used local diagnostic criteria for PDM and GDM (3, 32, 34, 40), and 17 studies did not address criteria for diabetes diagnosis at all. Evaluation of the differences between types of diabetes and its relation to functional cardiac changes in the offspring was not possible. Seventeen studies made a distinction between types of diabetes, while 14 studies were not consistent in the selection of diabetes types when comparing cases to controls in relation to functional cardiac changes (3–5, 9, 27, 30, 32–34, 39–42, 44).

Of the 1,679 cases identified in the review, only 100 (6%) infants were born to mothers with T1DM and 28 (1,7%) infants were born to mothers with T2DM. The majority of infants, 1,250 (74%), were born to mothers with GDM, while in 301 (18%) infants the type of maternal diabetes was not specified. The total number of cases used for different types of diabetes are summarized in Supplementary Table S2.

### 3.4 Risk of bias

Points awarded to each study by NOS are summarized in Table 2. The representativeness of infants born to mothers with diabetes (IDMs) was appropriately described in 29 of 31 studies and the controls were selected from the same hospitals as the cases in 29 of 31 studies. The echocardiographic measures used were reported in all 31 studies. Eighteen studies reported maternal HbA1c and/or diabetes therapy. Analysis of echocardiography was blinded in 12 studies (1, 4, 6, 7, 25, 28, 31, 37, 38, 41, 43, 44). Five studies adjusted for potential confounders such as race, birth weight, maternal age and time elapsed from delivery to postnatal outcome acquisition (3, 25, 41, 43, 44). IDMs were comparable with the control group with respect to postnatal age at outcome acquisition in all studies. Twenty-eight studies were scored as high quality, and three studies were scored as high risk of bias. No studies scored as very high risk of bias. A random effects model was used to incorporate heterogeneity in meta-analysis. Funnel plots based on IVSd and LVEF metanalysis were symmetric but with horisontal scatter, suggesting that the heterogeneity fit with the assumptions of the model, and raising no major concern about publication bias (54). The sources of heterogeneity could not be discerned from the funnel plots. Funnel plots are included as Supplementary Figures S1 and S2.

### 3.5 Cardiac function outcomes

The most reported echocardiographic parameter within each cardiac function category are summarized according to postnatal age in Tables 3, 4. The calculations used for summarized conclusions are available in Supplementary Table S7. Increased IVSd, decreased LVEF, increased MPI, decreased LV E/A ratio and/or increased PAP indicate abnormal cardiac function.

**Table 3:** Overall conclusion on cardiac function according to postnatal age of the child.

Postnatal age	1st week					1–6 months					1–8 years				
Number of studies and cases															
Type of diabetes (UNS = unspecified)	Any	DM1	DM2	GDM	UNS	Any	DM1	DM2	GDM	UNS	Any	DM1	DM2	GDM	UNS
No. studies	23	6	4	20	5	4	2	0	4	0	7	2	1	3	1
No. cases	1,136	62	21	865	188	392	38		354		503	38	7	312	146
No. controls	2,663					454					1,564				
Cardiac function overall conclusion (DM any type)															
Hypertrophy (IVSd)	Increased					Increased					No difference				
Systolic function (LVEF)	No difference					No difference					No difference				
Global ventricular function (MPI)	Decreased (MPI↑)					Decreased (MPI↑)					No difference				
Diastolic function (LV E/A)	Decreased					No difference					No difference				
Pulmonary hemodynamics (PAP)	PAP increased					(No data)					(No data)				
PDA	Inconclusive					(No data)					(No data)				

Children born to mothers with diabetes in pregnancy compared to children born to healthy control mother.

**Table 4:** Echocardiographic outcomes measures. Summary of studies according to age at acquisition.

Study	LV and RV morphology (IVSd)	Systolic function (LVEF)	Overall cardiac function (MPI)	Diastolic function LV E/A	Pulmonary hemodynamics (PAP)	Open PDA (cases vs. controls)
1st week						
Samanth et al. (5)	↑	↓	↑			
Arslan et al. (26)	↑	↑	↑	↑		
Smith et al. (24)	↑					23/40 vs. 23/40
Falqui et al. (30)	↔	↔		↓		
Vela-Huerta et al. (35)	↑	↓			↑	18/22 vs. 15/21
Sobeih et al. (33)			↑	↓		
Bagheri et al. (29)	↑	↔		↑		
Iwashima et al. (1)	↑	↔				
Ghandi et al. (23)	↑	↔	↑	↑		
Schierz et al. (32)	↑	↔	↔	↔		
Zablah et al. (4)	↔					11/75 vs. 44/556
Vela-Huerta et al. (34)	↑					
Hăşmăşanu et al. (9)	↑					
Çimen and Karaaslan (27)	↑		↔	↔		
Arslan et al. (22)	↑	↔				
Al-Biltagi et al. (2)	↑		↑	↓	↑	
Katheria and Leone (40)	↔				↑	5/32 vs. 5/18
Kozák-Bárány et al. (3)				↔		
Vela-Huerta et al. (28)	↑	↔				
Mehta et al. (31)	↑			↓		
Di Bernardo et al. (38)	↔	↑		↑		4/116 vs. 1/101
Sonaglioni (3d) (36)	↑	↔		↑		
Ergenc et al. (37)	↑		↑	↓		
1–6 months						
Aguilera et al. (25)	↔	↔	↔	↔		
Samanth et al. (5)	↑	↔	↑	** **	** **	** **
Arslan et al. (26)	↔	↔	↔	↔		
Sonaglioni (1.5 m) (36)	↑	↔		↑		
1–8 years						
Do et al. (39)	↑					
Hoodbhoy et al. (41)	↔	↔	** **	↓		
Blais et al. (6)	** **	** **	** **	↔		
Lestari et al. (42)	↑		** **			
Rijpert et al. (7)	↔		** **	↔		
Jacquemyn (1y) (44)	↓	↓	** **	↔		
Jacquemyn (2y) (44)	↓	↓	** **	↑		
Li et al. (43)	↔	↔	↔	↔		
No. studies	32	20	12	22	3	5

Of note, outside of the most reported parameters used for formal synthesis of results, five studies reported on LV global strain by 2D speckle-tracking echocardiography (2, 5, 24, 25, 41), two of these also on RV global strain (5, 24). Both LV and RV global strain was reduced in IDMs compared to controls in the 1st week of life and at 1–6 months.

#### 3.5.1 1st week of life

Of 20 studies that reported on the primary outcome category of morphology/hypertrophy, the majority (16 studies) found IVSd to be significantly increased in IDMs compared to controls. For systolic function (LVEF) no difference was found in seven out of 11 studies, two studies reported a decrease, while two studies reported an increase in LVEF comparing IDMs to controls. Eight studies reported on global ventricular function and of those, two studies found no difference, and six studies reported an increase in MPI comparing IDMs to controls. Five out of 11 studies found diastolic function impairment in IDMs compared to controls. In another five studies diastolic function was increased, and in three studies no difference was found in LV E/A ratio. All three studies reporting on pulmonary artery pressure found PAP to be increased within the first week of life.

Five studies reported on PDA status. Out of 285 cases, 61 (21,4%) had a PDA, and out of 736 controls, 88 (11,9%) had a PDA within the first 48 h of life. All PDA's were reported closed after 72 h of life with an additional scan (4, 24, 35, 38, 40).

#### 3.5.2 1–6 months

Two out of four studies found no difference in the primary outcome category of morphology/hypertrophy, whilst the two studies who did found an increased IVSd in IDMs compared to controls were of a higher number of participants. No difference was reported in systolic or diastolic function in any of the four studies. For global ventricular function one study found an increased MPI, whilst two studies found no difference comparing IDMs to controls. The one study with increased MPI had a greater number of cases and controls compared to the two studies which found no difference. LV E/A ratio was of no difference in 2 out of three studies, whilst one study found diastolic function to be increased in IDMs compared to controls.

#### 3.5.3 1–8 years

Three out of seven studies reported no difference regarding the primary outcome category of morphology/hypertrophy, two studies found increased IVSd, while the last two studies found no difference. Four studies reported echocardiographic measurements in systolic function by LVEF, two studies found no difference comparing children of diabetic mothers to controls, and two studies found a decreased in LVEF. One study measured global ventricular function by MPI and found no difference comparing cases to controls. Four out of six studies found no difference in diastolic function by LV E/A ratio, while one found a decreased LV E/A ratio, and one study found an increased LV E/A ratio comparing children of diabetic mothers to controls.

### 3.6 Meta-analysis

**Figure 2:**
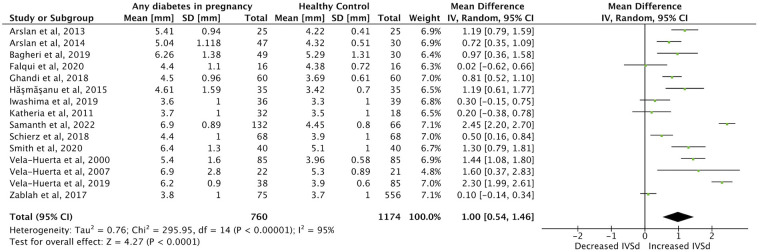
Forest plot displaying findings from 14 studies in meta-analysis of difference in interventricular septal thickness at end-diastole (IVSd) (mm) between infants born to mothers with diabetes (any type) and controls in the first week of life.

**Figure 3:**
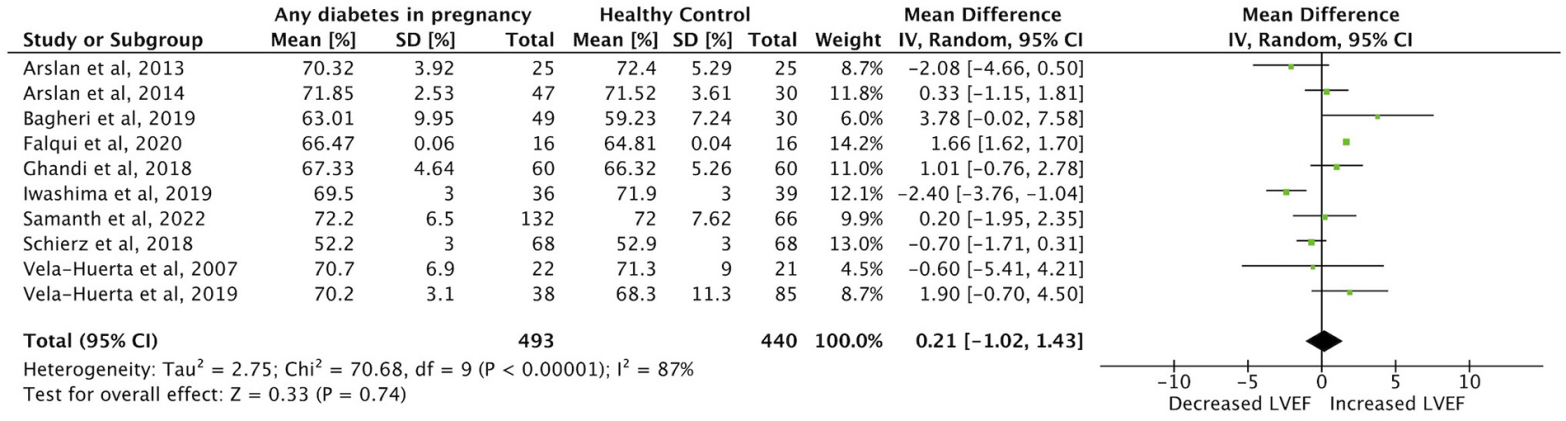
Forest plot displaying findings from eight studies in meta-analysis of difference in left ventricular ejection fraction (LVEF) (%) between infants born to mothers with diabetes (any type) and controls in the first week of life.

**Figure 4:**
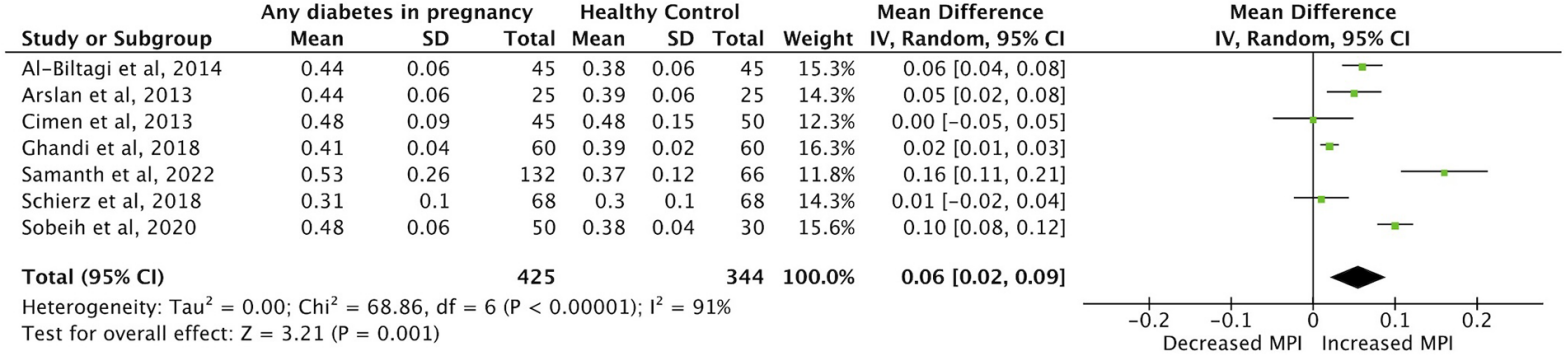
Forest plot displaying findings from nine studies in meta-analysis of difference in myocardial performance index (MPI) between infants born to mothers with diabetes (any type) and controls in the first week of life.

**Figure 5:**
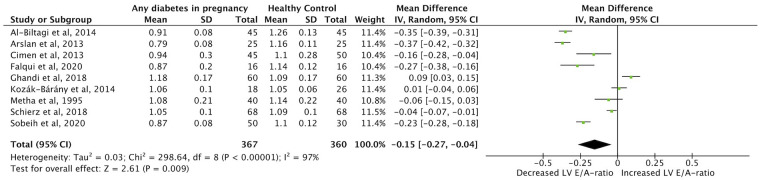
Forest plot displaying findings from nine studies in meta-analysis of difference in left ventricular E/A ratio (LV E/A) between infants born to mothers with diabetes (any type) and controls in the first week of life.

Meta-analysis was possible for the primary outcome category of IVSd, and for LVEF, LV E/A-ratio and MPI in the first week of life. Twenty studies were included in the meta-analysis (1–5, 9, 22–24, 26–35, 40). Forest plots comparing each outcome between IDMs and healthy control mothers were made (Figures 2–5). IVSd was significantly increased in IDMs compared to controls [mean difference 1.0 mm (95% CI, 0.54–1.46 mm)]. LVEF in IDMs was similar to controls [mean difference 0.2% (95% CI, −1.0 to 1.4%)]. MPI was significantly higher in IDMs than in controls [mean difference, 0.06 (CI 95%, 0.02–0.09)]. LV E/A ratio was significantly decreased in IDMs compared to controls [mean difference −0.15 (CI 95%, −0.27 to −0.04)]. In the analysis of all four parameters considerable heterogeneity between studies was found by I^2^ statistic.

### 3.7 Comparison to established normative values (Z-scores)

The absolute echocardiographic measurements (Supplementary Table S4) for IDMs compared to the example Z-score means calculated in the methods section were: In the first week of life: IVSd Z > 0 in 14/17 studies, LV E/A Z < 0 in 12/12 studies, MPI Z > 0 in 4/5 studies, LVEF in the normal range in 10/11 studies. At 1–6 months: IVSd Z > 0 in 5/5 studies, LV E/A Z < 0 in 1/3 studies, MPI Z > 0 in 2/2 studies, LVEF in the normal range in 5/5 studies. At 1–8 years: IVSd Z > 0 in 2/6 studies, LV E/A Z < 0 in 1/5 studies, MPI Z > 0 in 2/2 studies, LVEF in the normal range in 2/4 studies.

## 4 Discussion

### 4.1 Summary of evidence

This systematic review summarizes the association between maternal diabetes and altered cardiac function in offspring assessed by echocardiography. Based on observational studies comparing IDMs and control infants in the first week of life there is an association between maternal diabetes and the primary outcome category of morphology/myocardial hypertrophy (increased IVSd), as well as impaired diastolic function (decreased LV E/A), impaired global ventricular function (increased MPI), and impaired pulmonary hemodynamics (increased PAP and PDA). No change was found in systolic function (LVEF). The summarized conclusions on IVSd, LV E/A, MPI, and LVEF in the first week of life were confirmed in the meta-analysis. Results on cardiac function after the first week of life are based on comparatively small datasets and must therefore be interpreted with caution. For children aged 1–6 months there was an association between maternal diabetes and cardiac function assessed by ultrasound for the primary outcome of morphology/hypertrophy measured by increased IVSd as well as for MPI. No studies reported data for children from 6 months up to 1 years. For children from 1 to 8 years there was no difference between groups in IVSd or MPI, LVEF or LV E/A ratio.

Increased IVSd is rare in infants from normal pregnancies (9, 23, 34), but cardiomyopathy and septal hypertrophy has previously been reported to be present in about 30% of IDMs born with cardiac function abnormalities (2). The pathophysiology of maternal diabetes in relation to cardiac function of the infant is multifactorial and complex, and relates to the maternal glycaemic control (55). One of the main effects of maternal diabetes is the hyperglycaemic environment which leads to foetal and neonatal hyperinsulinism. Postnatal hyperinsulinism is usually a transient event for IDMs (56). This may explain why IVSd was significantly increased in both the meta-analysis and the overall conclusions of cardiac function in IDMs in the first week of life, but not later in life, a finding which is supported by a previous review of the role of hyperinsulinism in neonatal cardiac hypertrophy (56).

MPI is an indicator of overall cardiac performance including both systolic and diastolic function (2, 23). In our meta-analysis MPI was found to be higher in cases than in controls indicating a global worsening of the ventricular function. The clinical utility for MPI in childhood has not been fully established, and caution must be taken into consideration when characterizing MPI. The interpreting of MPI changes is loading dependent, as MPI increases with increased afterload and reduced preload. Still, long isovolumic phases generally imply worse function of the heart. High MPI at birth can decrease and normalize with maturation and reduced afterload over time, as seen in neonates born to a mother without diabetes (57). Samanth et al. found that MPI in infants of poorly controlled diabetic mothers was significantly higher than in infants of well controlled diabetic mothers indicating a correlation between the maternal glycaemic control and the ventricular function in IDMs. However, Al-Biltagi et al., did not find this correlation between increased MPI and maternal HbA1c.

LVEF was not found to be different for IDMs compared to controls. LVEF is very dependent on preload and afterload. In heart failure, LV dysfunction may frequently be identified by markers of deformation, when LVEF is normal. EF is further influenced by ventricular geometry, and increased wall thickness may contribute to preserved EF despite reduced longitudinal and circumferential shortening (58). MPI, stroke volume and cardiac output, together with indices of myocardial deformation, are likely to be more reliable quantifiers of LV systolic function.

Decreased mitral E/A ratio was seen for IDMs in their first week of life and was significantly decreased compared to healthy controls in the meta-analysis. Diastolic dysfunction in infants involves a reduction of the E wave and an increase of the A wave contributing to the inversion of the E/A ratio. The result is a value lower than 1, and could represent the first manifestation of diabetic cardiomyopathy (30). Even for women with good glycaemic control LV E/A ratio is found be decreased in IDMs (23, 30). The E/A waves are influenced by several factors including age, preload, afterload, and heart rate. In adults E/A is used as an indicator in the context of heart failure, its role as function index in children and neonates awaits further clarification (57). We were unable to investigate an association between glycaemic control and LV E/A ratio in this review due to lack of data.

PAP and PAAT were investigated as markers of pulmonary hemodynamics. PAAT was only reported in one study and was not included in our analysis. PAP can be estimated from tricuspid regurgitant flow velocity and shunt flows through ductus arteriosus (24). The pulmonary hemodynamics can be complicated by the presence of a PDA and/or atrial shunting. Increased atrial flow from left to right may suggest increased pulmonary flow if pulmonary pressures are normal (40). Increased PAP was found for IDMs within the first four days of life. PAP decreased to normal values in all cases at 72 h of life, but it was seen that the normalization was slower in cases than in controls (2, 35, 40). This transient disturbance in the adaptation of pulmonary circulation postnatally may be related to hypoglycemia in IDMs, delayed pulmonary maturation with respiratory morbidity and myocardial hypertrophy.

A delayed decrease in PAP may be contributed to by an increase of C-peptide concentration and beta-cell hyperplasia as seen in macrosomic IDMs. High leptin levels in the umbilical cord could also delay a fall in PAP, as leptin is positively associated with birthweight, indicating that macrosomic IDMs have a delayed normalization of the hemodynamic balance due to leptin-increased sympathetic activity in the kidneys and stimulation of the vascular smooth muscle proliferation (35).

PDA was more frequent in cases (21,4%) compared with controls (11,9%) and persisted longer in cases. Usually, a PDA closes within the first 12 h of life, but for IDMs PDA was frequently registered open until 72 h of life. Persistence of the ductus arteriosus could also contribute to the delayed transition of pulmonary hemodynamics and changes in myocardial compliance caused by an alteration of diastolic filling with an increased intraventricular pressure due to increased blood flow to the pulmonary circulation and left atrium.

Outside of the most reported parameters used for formal synthesis of results, myocardial strain was assessed in five studies which found reduced LV and RV global strain in IDMs compared to controls. Strain measures myocardial deformation, and varies with age, sex and loading conditions of LV and RV (5).

Comparing absolute echocardiographic measurements in IDMs to established normative values (Z-scores) in the first week of life we found IVSd Z > 0, LV E/A Z < 0 and MPI Z > 0 for the majority of studies. At 1–6 months we found IVSd Z > 0, and MPI Z > 0 for the majority of studies. At 1–8 years only MPI Z > 0. For all age groups LVEF was primarily in the normal range. Thus, comprison with example Z-scores for each age group supported the overall conclusions on cardiac function according to postnatal age in Table 3.

### 4.2 Strengths and limitations

#### 4.2.1 Strengths

The comprehensive search strategy of this review was structured and implemented in four different databases. Prior to the study, a protocol was registered, minimizing the risk of reporting bias. Additionally, screening and selection of studies, extraction of data, and assessment of risk and bias was accomplished by two reviewers independently. The data was presented according to the PRISMA guidelines.

#### 4.2.2 Limitations

Limitations to this review include the heterogeneity between studies due to different international definitions of GDM, different diagnostic criteria, lack of information on the degree of maternal glycaemic control and clinical treatment. Only five studies reported adjustments for different potential confounders (small sample size, gestational age, mode of delivery, race, maternal age, time elapsed from delivery to postnatal visit, and change in infant weight from birthweight), which can lead to a wrong estimation of the observed association between diabetes in pregnancy and investigated outcomes. Other potential confounders not adjusted for in any of the studies include, but are not limited to, maternal comobidity, smoking, maternal BMI and levels of HbA1c during pregnancy.

Grouping of cases and outcome reporting was heterogeneous across studies. E.g., Çimen et al. had two case groups of IDMs. Group 1 had an interventricular septum thickness greater than normal, and group 2 had an interventricular septum thickness normal in size. Applied normal ranges for thickness of the interventricular septum is another factor to be considered. E.g., Çimen et al., defined “septal hypertrophy” as IVSd more than 5 mm, while Arslan et al. used 6 mm as the limit for septal hypertrophy. With regards to understanding effects of maternal diabetes on cardiac function a major limitation is the inability of the reported echocardiographic parameters to pick up more subtle cardiac dysfunction. E.g., myocardial strain and strain rate analysis may identify clinically significant myocardial dysfunction despite normal LVEF, MPI and E/A ratio. Equally, right heart function was not directly addressed in this review. With the effects of maternal diabetes on the pulmonary circulation and offspring respiratory function, altered right ventricle function may be more important than that of the left ventricle.

The potential sources of heterogeneity observed in the meta-analysis include the type of diabetes, duration of the diabetes and treatment, image acquisition, inter-person scan and analysis variability and more.

Comparatively few studies examined offspring cardiac function after the first week of life. Counting the total number of cases and controls, data included for the two oldest age groups were 4,1 times (1–6 months) and 1,6 times (1–8 years) lower in quantity than for the first week of life. The majority of diabetes cases were of GDM, which in most situations are a transient event for the women. Whereas T1DM and T2DM are chronic conditions. With only a few percent of reported cases being born to mothers with T1DM or T2DM, there is a lack of data on these infants, and the question still remains if there is a difference in the long-term outcome in the children regards to which type of diabetes their mother had. A major limitation when interpreting the results is that the available data did not allow for sub-analysis based on the type of diabetes, treatment, or glycemic control. This highlights that future studies should analyze GDM and PDM subtypes separately and document treatment and glycemic control.

The biggest population of cases and controls was the Chinese study from Li et al. including only GDM, which contributed 230 cases in the group of 2–8 years old's. The amount of people diagnosed with GDM can differ from different countries depending on which criteria is used for inclusion. China is using the same criteria as WHO recommendations which compared to criteria used in e.g., Scandinavia is far from the same. This means that more people will be diagnosed with GDM in China than in Scandinavia leading to an uneven distribution of cases.

### 4.3 Conclusion

Diabetes in pregnancy is associated with offspring cardiac hypertrophy, the primary outcome category in this systematic review, and with impaired diastolic and global cardiac function in infants up to one week old. Most of the studies including older children found either no difference or had small numbers of cases and control. There is very little data on pre-gestational diabetes, which may have significantly different impact on offspring cardiac function compared with GDM. Further, there is little to no information on right ventricle function, which may be where the most important short- and long-term effects are seen. Limitations to the current evidence means that more knowledge is required to understand the association between maternal diabetes in pregnancy and long-term cardiac health. To develop strategies to mitigate the risk of future cardiovascular complications we need longitudinal studies with larger sample sizes and more accurate information on diagnostic criteria, maternal diabetes type, diabetes therapy and glycaemic control. Better glycemic regulation and optimized medical treatment of mothers with diabetes will improve infant outcomes, however there is likely an effect of maternel diabetes regardless of glycemic control. Some studies point towards insulin as a metabolic effector targeting the cardiomyocytes and fetal echocardiography to screen for possible birth defects is advised regardless of the type of diabetes. Maternel diet, tailored antihypertensive treatment and patient education may further improve pregnancy outcomes. We still need to learn more about infant cardiac function, metabolic status, growth and possible modifiable factors after birth. Applying more advanced echocardiographic measures or cardiac MRI may detect subtle changes with potential important long-term consequences, e.g., in myocardial remodeling and contractility. Increasing our knowledge on cardiovascular health in infants born to women with diabetes is important to improve pregnancy care, and to tailor the best possible follow up for these children.

## Data Availability

The original contributions presented in the study are included in the article/**Supplementary Material**, further inquiries can be directed to the corresponding author.
