# Supplementary material for: Diabetes in pregnancy and offspring cardiac function: a systematic review and meta-analysis
Source: Front Pediatr. 2024 Jul 18;12:1404625. doi: 10.3389/fped.2024.1404625 (PMC11291373; doi:10.3389/fped.2024.1404625)

Diabetes in Pregnancy and Offspring Cardiac Function: A Systematic Review and Meta-analysis

Supplementary Material

1 Supplementary Figures and Tables

Supplementary Table 1. Search strategy

PubMED:

1. “Infant” [Mesh]
2. “infant*”[Title/Abstract]
3. “neonatal” [Title/Abstract]
4. “newborn*” [Title/Abstract]
5. “baby”* [Title/Abstract]
6. “neonates” [Title/Abstract]
7. “child” [Mesh]
8. “child”* [Title/Abstract]
9. “Diabetes Mellitus” [Mesh]
10. “diabetes” [Title/Abstract]
11. “gestational diabetes” [Title/Abstract]
12. “pregestational diabetes” [Title/Abstract]
13. “Heart Function Tests” [Mesh]
14. “cardiac function” [Title/Abstract]
15. “heart function” [Title/Abstract]
16. “myocardium” [Title/Abstract]
17. “echocardiography” [Title/Abstract]
18. #1 OR #2 OR #3 OR #4 OR#5 OR #6 OR #7 OR #8
19. #9 OR #10 OR#11 OR #12
20. #13 OR #14 OR#15 OR #16 OR #17

21. #18 AND #19 AND #20

Search founded 02.02.22, updated 26.06.23

Articles included from 1992-2022

796 titles

EMBASE:

1. “infant”/exp
2. “infant”* ab,ti
3. “neonatal” ab,ti
4. “newborn” ab,ti
5. “baby”* ab,ti
6. “neonate”* ab,ti
7. “child”/exp
8. “child”* ab,ti
9. “diabetes mellitus”/exp
10. “diabetes” ab,ti
11. “pregnancy diabetes mellitus” ab,ti
12. ”pregestational diabetes mellitus” ab,ti
13. ”heart function”/exp
14. ”heart function”* ab,ti
15. ”cardiac muscle” ab,ti
16. ”echocardiography” ab,ti
17. #1 OR #2 OR #3 OR #4 OR#5 OR #6 OR #7 OR #8
18. #9 OR #10 OR#11 OR #12
19. #13 OR #14 OR#15 OR #16
20. #17 AND #18 AND #19

Search founded 02.02.22, updated 26.06.23

Articles included from 1992-2022

Excluded: Conference abstracts, case-rapports

Only human studies

Limited to (age): Newborn (0-1 month), infant (1-12 month), child (1-12 years), adolescent (13-17 years), adult (18-64 years), young adult (18-24 years)

Limited to (diseases): Cardiomyopathy, cardiovascular disease, congenital heart disease, diabetes mellitus, diabetic cardiomyopathy, diabetic ketoacidosis, heart arrhythmia, heart disease, heart failure, heart infarction, heart left ventricle hypertrophy, heart ventricle hypotrophy, heart ventricle septum defect, hyperglycemia, hypertrophic cardiomyopathy, hypoglycemia, insulin dependent diabetes mellitus, tachycardia, insulin resistance, maternal diabetes mellitus, pregnancy diabetes mellitus.

1212 titles

Cochrane:

1. “infant” [Mesh]
2. “infant” ti;ab;kw
3. “neonatal” ti;ab;kw
4. “neonate” ti;ab;kw
5. “newborn” ti;ab;kw
6. “child” [Mesh]
7. “child” ti;ab;kw
8. “diabetes mellitus” [Mesh]
9. “diabetes” ti;ab;kw
10. “diabetes mellitus” ti;ab;kw
11. “gestational diabetes” ti;ab;kw
12. “pregestational diabetes” ti;ab;kw
13. ”heart function tests” [Mesh]
14. “heart function” ti;ab;kw
15. ”cardiac function” ti;ab;kw
16. “myocardium” ti;ab;kw
17. “echocardiography” ti;ab;kw
18. #1 OR #2 OR #3 OR #4 OR #5 OR #6 OR #7

19. #8 OR#9 OR #10 OR #11 OR #12
20. #13 OR#14 OR #15 OR #16
21. #18 AND #19 AND #20

Search founded 22.02.22, updated 26.06.23

Articles included from 1992-2022

229 titles

Web of Science:

1. “infant”(Topic)
2. “neonatal” (Topic)
3. “newborn” (Topic)
4. “baby” (Topic)
5. “neonate” (Topic)
6. “child” (Topic)
7. “diabetes mellitus” (Topic)
8. “diabetes” (Topic)
9. “gestational diabetes” (Topic)
10. ”pregestational diabetes” (Topic)
11. ”heart function” (Topic)
12. ”cardiac function” (Topic)
13. ”myocardium” (Topic)
14. ”echocardiography” (Topic)
15. #1 OR #2 OR #3 OR #4 OR#5 OR #6
16. #7 OR#8 OR #9 OR #10
17. #11 OR#12 OR #13 OR #14
18. #15 AND #16 AND #17

Search founded 22.02.22, updated 26.06.23

Articles included from 1992-2022

Only studies within pediatrics, cardiology, general health care or obstetrics

512 titles

The final search results of PubMED, EMBASE, Cochrane and Web of Science were compiled in EndNote 20 (Clarivate, 2013, Philadelphia, PA. Available at www.endnote.com) for duplication removal, then Covidence Systematic Review Software (Veritas Health Innovation, 2022, Melbourne, Australia. Available at www.covidence.org) was used for screening for inclusion and data extraction.

Supplementary Table 2. Total number of echocardiographies in cases and controls and criteria used for diabetes diagnosis

Studies	Cases total	DM type 1	DM type 2	GDM	DM type not specified	Controls	All participants	Criteria used for DM or GDM diagnosis
**1st week**								
Arslan 2013	25			25		25	50	Not specified
Samanth (at birth) 2021	132	19		113		66	198	ADA
Smith 2020	40			40		40	80	Not specified
Falqui 2020	16	2	2	11	1	16	32	Not specified
Vela-Huerta 2007	22			22		21	43	ADA
Sobeih 2020	50			41	9	30	80	Not specified
Bagheri 2019	49		5	44		30	79	Not specified
Iwashima 2019	36				36	39	75	Not specified
Ghandi 2018	60			60		60	120	WHO
Schierz 2018	68	10	6	52		68	136	INIH
Zablah 2017	75	2	8	60	5	556	631	Not specified
Vela-Huerta 2000	85			74	11	85	170	Not specified
Hăşmăşanu 2015	35			24	11	35	70	Not specified
Cimen 2013	37				37	50	87	Not specified
Arslan 2014	47			47		30	77	Not specified
Al-Biltagi 2014	45	20		25		45	90	WHO
Katheria 2011	32				32	18	50	ACOG
Kozák-Bárány 2004	18	9		9		26	44	Not specified
Vela-Huerta 2019	38				38	85	123	ADA
Metha 1995								
→ LGA	16			16		17	33	Not specified
→ AGA	24			24		40	64	Not specified
Bernardo 2022	116			116		101	217	ADA
Sonaglioni (3d) 2022	30			30		30	60	Not specified
Ergenc 2023	32			32		22	54	ADA
**1-6 months**								
Arslan 2013	25			25		25	50	Not specified
Samanth 2021								

→ 6 months	132	19		113		66	198	ADA
→ 6 weeks	132	19		113		66	198	ADA
Aguilera 2020	73			73		73	146	NICE
Sonaglioni (1.5m) 2022	30			30		30	60	Not specified
**1-8 years**								
Do 2021	25				25	20	45	Not specified
Hoodbhoy 2018	68	8	7	53		68	136	Not specified
Blais 2018	65			29	36	41	106	Not specified
Lestari 2018	23				23	23	46	WHO
Rijpert 2011	30	30				30	60	Not specified
Jacquemyn 2023								
→ 1 year	37				37	40	77	Not specified
→ 2 year	25				25	20	45	Not specified
Li 2022	230			230		864	1094	ADA
**Total Echocardiographies**	**2023**	**138**	**28**	**1531**	**326**	**2901**	**4924**	
**Total infants**	**1679**	**100**	**28**	**1250**	**301**	**2694**	**4373**	

ADA: American Diabetes Association. ACOG: American College of Obstetricians and Gynecologists. NICE: National Institute for Health and Care Excellence. INIH: The Italian National Institute of Health DM: Diabetes Mellitus. GDM: Gestational diabetes. LGA: Large for gestational age. AGA: Average for gestational age.

Supplementary Table 3. Treatment of diabetes and glycemic control

Study	Treatment of diabetes				Glycaemic control (HbA1c)							
	Diet	Oral meds	Insulin	Combi	DM1		DM2		GDM		Type not specified	
					Mean	SD	Mean	SD	Mean	SD	Mean	SD
**1st week**												
Arslan 2013									5,6	0,84		
Samanth (at birth) 2021												
Smith 2020	21		19									
Falqui 2020			16								5,7	1
Vela-Huerta 2007	2		20									
Sobeih 2020												
Bagheri 2019			23									
Iwashima 2019												
Ghandi 2018	33		27						5,38	0,42		
Schierz 2018	68		22						5,7		6,6	
Zablah 2017	29	17	23								6,79	1,17
Vela-Huerta 2000									10,40	1,73	10,16	2,55
Hăşmăşanu 2015			4									
Cimen 2013												
Arslan 2014												
Al-Biltagi 2014												
Katheria 2011												
Kozák-Bárány 2004	2		16		6,41	0,64			5,5	0,25		
Vela-Huerta 2019									10,40	1,73	10,16	2,55
Metha 1995												
Bernardo 2022									5,2			
Sonaglioni (3d) 2022	16		14						5,3	2,5		
Ergenc 2023			15									
**1-6 months**												
Arslan 2013									5,6	0,84		
Samanth (6m) 2021												
Samanth (6w) 2021												
Aguilera 2020	23	24	10	16					5,5	0,4		
Sonaglioni (1.5m) 2022												

**1-8 years**												
Do 2021												
Hoodbhoy 2018	24	25	18									
Blais 2018												
Lestari 2018												
Rijpert 2011					6,29	0,84						
Jacquemyn (1y) 2023												
Jacquemyn (2y) 2023											6,4	
Li 2022												
**Total**	**218**	**66**	**227**	**16**	**6,35**				**6,46**		**7,65**	

Supplementary Table 4. All extracted echocardiographic outcome measurements in each age group

Study	Morphology (IVSd (mm))										Systolic function (LVEF)									
	DM1		DM2		GDM		Type not specified		Control		DM1		DM2		GDM		Type not specified		Control	
	Mean	SD	Mean	SD	Mean	SD	Mean	SD	Mean	SD	Mean	SD	Mean	SD	Mean	SD	Mean	SD	Mean	SD
**1st week**																				
Arslan 2013					5,41	0,94			4,22	0,41					70,32	3,92			72,4	5,29
Samanth (at birth) 2021							6,83	0,72	4,45	0,8							72,39	6,32	71,98	7,62
Smith 2020					6,4	1,3			5,1	1										
Falqui 2020							4,4	1,1	4,38	0,72							66,48	0,06	64,81	0,04
Vela-Huerta 2007					6,9	2,8			5,3	0,89					71,6	7,35			71,55	9,25
Sobeih 2020																				
Bagheri 2019							6,26	1,38	5,29	1,31							63,01	9,95	59,23	7,24
Iwashima 2019					3,6				3,3						69,5				71,9	
Ghandi 2018					4,5	0,96			3,69	0,61					67,33	4,64			66,32	5,26
Schierz 2017					4,2		5		3,9						49,8		53,45	57,5		
Zablah 2017							3,8	0,3	3,7	0,35										
Vela-Huerta 2000							5,66	1,27	5,49	0,605										
Hăşmăşanu 2015							4,61	1,59	3,42	0,7										
Cimen 2013																				
Arslan 2014					5,36	0,52			4,32	0,51					71,89	2,69			71,52	3,61
Al-Biltagi 2014																				

Katheria 2011							3,7		3,5											
Kozák-Bárány 2004																				
Vela-Huerta 2019																				
Metha 1995																				
Bernardo 2022					3,6				3,5						68				65	
Sonaglioni 2022					3,9	0,5			3,3	0,2					71,6	2,9			71,9	2,8
Ergenc					6				5											
**1-6 months**																				
Arslan 2013					4,8	0,5			4,32	0,51					73,28	4,74			72,52	4,62
Samanth (6m) 2021							6,11	1,14	4,48	0,8							71,07	6,21	70,87	7,12
Samanth (6w) 2021							6,55	0,64	4,12	0,76							71,3	6,09	70,18	9,54
Aguilera 2020					4,2	0,37			4,8	0,35					67,2	4,48			69,4	2,47
Sonaglioni 2022					4,2	0,3			3,7	0,4					71,1	3,1			71,7	2,5
**1-8 years**																				
Do 2021							1,2	0,27	0,5	0,25										
Hoodbhoy 2018							4	0,38	4	0,38							65	1,74	66,1	1,25
Blais 2018																				
Lestari 2018							6,5	0,5	5,8	0,7										
Rijpert 2011																				
Jacquemyn (1y) 2023							4,0		4,41								52,0		63,0	
Jacquemyn (2y) 2023							4,0		4,78								54,0		63,0	
Li 2022					3,81	0,58			3,81	0,55					66,56	4,11			67,00	3,88

Study	Global ventricular function (MPI/Tei-index)										Diastolic function (LV E/A)									
	DM1		DM2		GDM		Type not specified		Control		DM1		DM2		GDM		Type not specified		Control	
	Mean	SD	Mean	SD	Mean	SD	Mean	SD	Mean	SD	Mean	SD	Mean	SD	Mean	SD	Mean	SD	Mean	SD
**1st week**																				
Arslan 2013															0,79	0,08			1,16	0,11
Samanth (at birth) 2021							0,53	0,23	0,37	0,12										
Smith 2020																				
Falqui 2020																	0,87	0,2	1,14	0,12
Vela-Huerta 2007																				
Sobeih 2020							0,48	0,06	0,38	0,04							0,87	0,08	1,1	0,12
Bagheri 2019																				
Iwashima 2019																				
Ghandi 2018															1,18	0,17			1,09	0,17
Schierz 2017					0,285		0,32		0,305						1,05		1,05		1,09	
Zablah 2017																				
Vela-Huerta 2000																				
Hăşmăşanu 2015																				
Cimen 2013																				
Cimen 2013																				
Arslan 2014																				
Al-Biltagi 2014	0,45	0,06			0,43	0,05			0,38	0,06	0,89	0,07			0,92	0,08			1,26	0,13

Katheria 2011																				
Kozák-Bárány 2004																	1,06	0,1	1,05	0,06
Vela-Huerta 2019																				
Metha 1995															1,01	0,13			1,14	0,205
Metha 1995															1,13	0,23			1,14	0,219
Bernardo 2022															1,08				1,00	
Sonaglioni 2022															0,9	0,2			0,7	0,2
Ergenc					0,57				0,46						0,83				1,28	
**1-6 months**																				
Arslan 2013															1,26	0,17			1,28	0,12
Samanth (6m) 2021							0,43	0,17	0,38	0,12										
Samanth (6w) 2021							0,47	0,17	0,38	0,12										
Aguilera 2020															1,3	0,22			1,4	0,26
Sonaglioni 2022															0,7	0,2			0,5	0,2
**1-8 years**																				
Do 2021																				
Hoodbhoy 2018							0,37	0,2	0,36	0,2							1,6	0,22	1,6	0,23
Blais 2018															1,83	0,43			1,93	0,52
Lestari 2018																				
Rijpert 2011											2,14	0,35							2,2	0,23
Jacquemyn (1y) 2023																	1,41		1,40	
Jacquemyn (2y) 2023																	1,68		1,51	
Li 2022					43,48	6,45			43,21	6,22					1,78	0,32			1,80	0,33

Study	Pulmonary Hemodynamics										PDA									
	DM1		DM2		GDM		Type not specified		Control		DM1		DM2		GDM		Type not specified		Control	
	Mean	SD	Mean	SD	Mean	SD	Mean	SD	Mean	SD	Mean	SD	Mean	SD	Mean	SD	Mean	SD	Mean	SD
**1st week**																				
Arslan 2013																				
Samanth (at birth) 2021																				
Smith 2020	PAAT				47	11			62	16					23/40				23/40	
Falqui 2020																				
Vela-Huerta 2007	PAP						35,5	8,45	30,85	7,25							18/22		15/21	
Sobeih 2020																				
Bagheri 2019																				
Iwashima 2019																				
Ghandi 2018																				
Schierz 2017	PAP systolic				10,925		17,4		12											
Zablah 2017																	11/75		44/556	
Vela-Huerta 2000																				
Hăşmăşanu 2015																				
Cimen 2013																				
Arslan 2014																				
Al-Biltagi 2015	PAP systolic				36,5	6	38,5	5	30,5	4										

Katheria 2011	PAP						34	12,1	33,7	10,4							5/32		5/18	
Kozák-Bárány 2004																				
Vela-Huerta 2019																				
Metha 1995																				
Bernardo 2022																				
Sonaglioni 2022																				
Ergenc																				
**1-6 months**																				
Arslan 2013																				
Samanth (6m) 2021																				
Samanth (6w) 2021																				
Aguilera 2020																				
Sonaglioni 2022																				
**1-8 years**																				
Do 2021																				
Hoodbhoy 2018																				
Blais 2018																				
Lestari 2018																				
Rijpert 2011																				
Jacquemyn (1y) 2023																				
Jacquemyn (2y) 2023																				
Li 2022																				

Supplementary Table 7. The calculations used for summarized conclusions in Tables 3 and 4 in the manuscript. **7A**. Summary of reported increase, decrease or no difference in cardiac function of infants born by mothers with diabetes compared with controls according to postnatal age of the child **7B**. Summary of cardiac function per study and outcome category.

Supplementary Table 7A. Summary of reported increase, decrease or no difference in cardiac function of infants born by mothers with diabetes compared with controls according to postnatal age of the child

		1st week			1-6 months			1-8 years		
		Number of studies (%)	n cases	n controls	Number of studies (%)	n cases	n controls	Number of studies (%)	n cases	n controls
**LV & RV morphology (hypertrophy)**	Increased	16 (80,0%)	675	1208	2 (62,3%)	162	228	2 (10,9%)	48	43
	Decreased							2 (14,2%)	62	60
	No difference	4 (20,0%)	239	814	2 (37,7%)	98	196	3 (74,9%)	328	962
	**Total**	**20 (100%)**	**914**	**2022**	**4 (100%)**	**260**	**424**	**7 (100%)**	**438**	**1065**
**Systolic function**	Increased	2 (23,5%)	141	151						
	Decreased	2 (25,6%)	154	241				2 (17,2%)	62	60
	No difference	7 (50,9%)	306	549	4 (100%)	260	424	2 (82,8%)	298	932
	**Total**	**11 (100%)**	**601**	**941**	**4 (100%)**	**260**	**424**	**4 (100%)**	**360**	**992**
**Global ventricular function**	Increased	6 (75,3%)	344	560	1 (57,4%)	132	198			
	Decreased									
	No difference	2 (24,7%)	113	281	2 (42,6%)	98	196	1 (100%)	230	864
	**Total**	**8 (100%)**	**457**	**841**	**3 (100%)**	**230**	**394**	**1 (100%)**	**230**	**864**
**Diastolic function**	Increased	5 (32,8%)	134	249	1 (23,4%)	30	30	1 (5,5%)	25	20
	Decreased	5 (35%)	143	224				1 (14,9%)	68	68
	No difference	3 (32,2%)	131	325	2 (79,6%)	98	196	4 (79,6%)	362	975
	**Total**	**13 (100%)**	**408**	**798**	**3 (100%)**	**128**	**226**	**6 (100%)**	**455**	**1063**
**PAP**	Increased	3 (100%)	99	84						

	Decreased									
	No difference									
	**Total**	**3 (100%)**	**99**	**84**						
**PDA**	Open	5 (14,6%)	61	88						
	Not open	5 (85,4%)	224	648						
	**Total**	**5 (100%)**	**285**	**736**						

Supplementary Table 7B. Summary of cardiac function findings by age group per study and outcome category.

**Study**	**Hypertrophy (IVSd)**	**Systolic function (LVEF)**	**Global ventricular function (MPI)**	**Diastolic function (LV/EA)**
		**1st week (23 studies)**		
Smith 2020	Increased			
Falqui 2020	No difference	No difference		Decreased
Vela-Huerta 2007	Increased	Decreased		
Sobeih 2020			Increased	Decreased
Bagheri 2019	Increased	No difference		Increased
Iwashima 2019	Increased	No difference		
Ghandi 2018	Increased	No difference	Increased	Increased
Schierz 2018	Increased	No difference	No difference	No difference
Zablah 2017	No difference			
Vela-Huerta 2000	Increased			
Hăşmăşanu 2015	Increased			
Cimen 2013	Increased		No difference	No difference
Arslan 2014	increased	No difference		
Al-Biltagi 2014	Increased		Increased	Decreased
Katheria 2011	No difference			
Kozák-Bárány 2004				No difference
Vela-Huerta 2019	Increased			

Mehta 1995				Decreased
Arslan 2013	Increased	Increased	Increased	Increased
Samanth 2021	Increased	Decreased	Increased	
Bernardo 2022	No difference	Increased		Increased
Sonaglioni 2022	Increased	No difference		Increased
Ergenc 2023	Increased		Increased	Decreased
**1-6 months (4 studies)**				
Arslan 2013	No difference	No difference	No difference	No difference
Samanth 2021	Increased	No difference	Increased	
Aguilera 2020	No difference	No difference	No difference	No difference
Sonaglioni 2022	increased	No difference		Increased
**2-8 years (8 studies)**				
Do 2021	Increased			
Hoodbhoy 2018	No difference	No difference		Decreaed
Blais 2018				No difference
Lestari 2018	Increased			
Rijpert 2011	No difference			No difference
Jacquemyn (1y) 2023	Decreased	Decreased		No difference
Jacquemyn (2y) 2023	Decreased	Decreased		Increased
Li 2022	No difference	No difference	No difference	No difference

Supplementary Figure 1. Funnel Plot IVSd

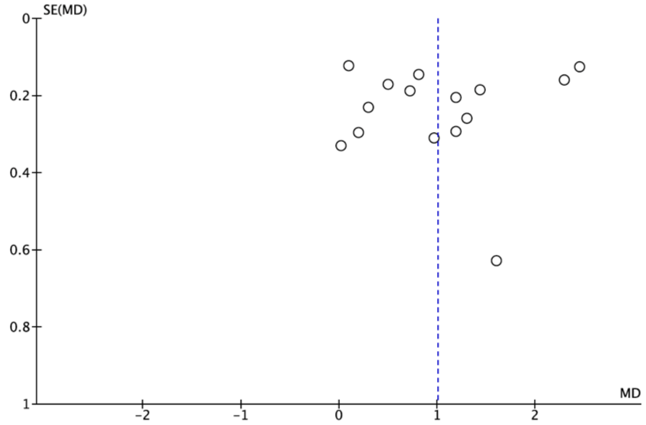

Supplementary Figure 2. Funnel Plot LVEF

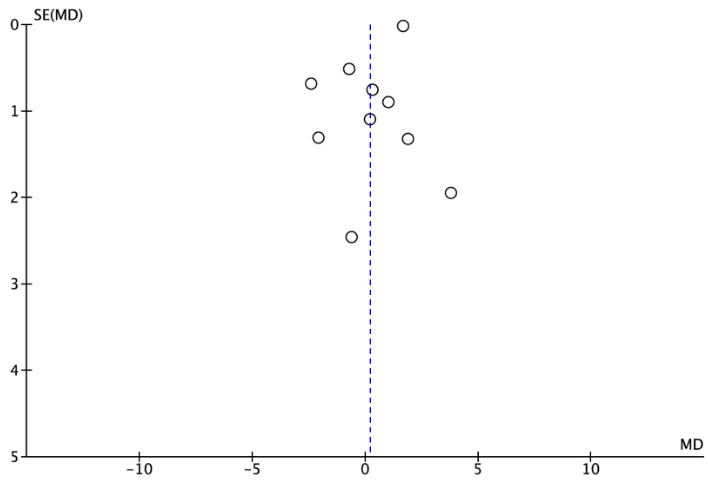

Supplement: Supplementary file 1 [file Datasheet1.pdf]
